# A content analysis of global and national policies, plans, and guidelines to integrate NCDs with HIV care in LMICs

**DOI:** 10.1371/journal.pgph.0007072

**Published:** 2026-08-07

**Authors:** Reet Kapur, Abby Briggs, Dina Moinul, Teona Giorgadze, Gloria Guevara Alvarez, Kellen Nyamurungi Namusisi, Jonathan Purtle, Mari Armstrong-Hough, Donna Shelley

**Affiliations:** 1 Department of Health Policy and Management, School of Global Public Health, New York University, New York, New York, United States of America; 2 Dartmouth Center for Implementation Science, Geisel School of Medicine, Hanover, New Hampshire, United States of America; 3 Environmental Health Sciences, Department of Medicine, New York University, New York, New York, United States of America; 4 Department of Epidemiology/Social and Behavioral Sciences, School of Global Public Health, New York University, New York, New York, United States of America; Qatar University College of Medicine, QATAR

## Abstract

The growing burden of non-communicable diseases (NCDs) among people living with HIV (PWH) in low- and middle-income countries (LMICs), threatens gains in life expectancy. Global organizations have called for integrating NCD management into existing HIV care systems, but it is unclear what actions countries are taking to accomplish this goal. To bridge this knowledge gap, we conducted a quantitative content analysis of HIV policy documents from the World Health Organization (WHO), the US President's Emergency Plan for AIDS Relief (PEPFAR) and 22 PEPFAR-sponsored LMICs to analyze mentions of: 1) screening and treatment of selected NCDs (hypertension, diabetes, cervical cancer, and depression) and/or two NCD risk factors (alcohol and tobacco use), and 2) health system-strengthening strategies when mentioned in relation to the selected NCDs and risk factors. We used the WHO’s Building Blocks framework to identify these strategies. Screening and treatment for cervical cancer and mental health conditions were reported in the WHO and PEPFAR documents and in the majority of country documents. Strategies to strengthen service delivery (e.g., NCD care coordination across health facilities, establishing guidelines for integrated care) were frequently referenced across WHO, PEPFAR, and country-level documents. Far fewer documents mentioned strategies related to health information technology, medication access, and financing to support care integration. Although the WHO, PEPFAR and country-level documents illustrate a commitment to NCD-HIV health system integration, there was variation in which NCDs were being prioritized. Mentions of screening and treatment of NCD risk factors were rare, suggesting that tobacco and alcohol-related services may be comparatively under-resourced. Integrating NCD and HIV care requires sustained investments that align with local resources and attention to system-level enablers (e.g., improved information systems, stronger medicine supply chains) that support comprehensive care. Future research should focus on identifying the determinants of policy adoption, including factors that either drive or constrain integration of the full spectrum of NCD services within HIV care settings.

## Introduction

The growing burden of non-communicable diseases (NCDs) among people living with Human Immunodeficiency Virus (PWH) is threatening gains in life expectancy achieved through increased access to antiretroviral therapy [1]. It is estimated that 20% of PWH have been diagnosed with at least one NCD, with low- and middle-income countries (LMICs) disproportionally impacted by these trends [2,3]. To address this challenge, the WHO has called for integrating the prevention and management of major NCDs in the context of HIV care to improve HIV treatment outcomes [4,5].

Evidence from high-income countries and LMICs shows that integrating HIV care with screening and/or treatment for NCDs and substance use disorders improves overall health outcomes for PWH, enhances health system efficiency and reduces healthcare costs [5–7]. The WHO, UN, UNAIDS, and the US President’s Emergency Plan for AIDS Relief (PEPFAR) all emphasize the importance of routine NCD screening, diagnosis, and treatment for PWH [5,8–13]. Moreover, PEPFAR has funded collaborative research initiatives such as the NCD/HIV Integrated Care Project (2014–2021) to advance this agenda [14].

NCD-HIV integration in LMICs is enabled by a range of factors that are outlined in the WHO’s six Building Blocks for health systems strengthening and are also endorsed by PEPFAR [13,15]. These factors include a trained workforce, health information systems, reliable medication supply chains, financing, and leaders who prioritize integration [7,16–20]. The WHO building blocks further serve as indicators to evaluate system robustness and to analyze whether the conditions necessary for integrated NCD-HIV care are currently being met in LMICs [15].

Despite strong global support and evidence in favor of integration, LMICs face persistent, systemic challenges in implementing evidence-based interventions for NCD screening and treatment within HIV care settings. These challenges include weak referral systems, vertical care delivery models, shortage of trained clinical staff, and a lack of consistent funding [5,17,21]**.** Currently, it remains unclear whether ongoing efforts by countries to advance NCD-HIV integration align with the WHO’s health system-strengthening strategies.

To bridge this knowledge gap, we conducted a content analysis of HIV policy documents from the WHO, PEPFAR and PEPFAR-sponsored LMICs to analyze mentions of: 1) screening and treatment of selected NCD and NCD risk factors within HIV care settings, and 2) health system-strengthening strategies, as defined by the WHO Building Blocks, when mentioned in relation to the selected NCDs and/or risk factors.

## Methods

### Document inclusion criteria

We reviewed documents published between 2010 and 2023, authored by World Bank-designated LMICs that are supported by PEPFAR [22]. We included three types of country-level HIV guidance documents: National Strategic Plans (NSPs), National Technical Guidelines (NTGs), and PEPFAR Country Operational Plans (COPs). To facilitate cross-country comparisons, only countries that had all three documents publicly available in English were included in the analysis. PEPFAR-supported countries were selected for this analysis because PEPFAR is the largest funder of HIV programs globally, and its policies drive HIV care in LMICs by setting data-driven targets for integrating best practices in local systems [13].

To compare LMIC’s national policies with global guidance, we also analyzed two documents published by global organizations: the 2021 WHO Consolidated Guidelines on HIV Prevention, Testing, Treatment, Service Delivery, and Monitoring, and the 2023 PEPFAR Country and Regional Operational Plan Guidance and Technical Considerations [23,24]. Table 1 describes each document type and its relevance to this analysis.

**Table 1:** Definition and scope of included global and country documents [25,26].

Document Type	Publisher	Definition and scope
**2021 WHO Consolidated Guidelines on HIV Prevention, Testing, Treatment, Service Delivery, and Monitoring**	World Health Organization	**Guidelines** consolidating clinical and programmatic recommendations across different populations and settings, incorporating all relevant WHO guidance on HIV produced since 2016.
**2023 Country and Regional Operational Plan Guidance and Technical Considerations**	PEPFAR/ US State Department	**Annual guidelines** outlining strategic, programmatic, and financial frameworks for PEPFAR-supported countries.
**National Strategic Plan (NSP)**	Country government or Ministry of Health	**Country-level plan** that outlines priorities, strategies, and actions for the management of HIV over a specified period.
**National Technical/Clinical Guidelines (NTG)**	Country government or Ministry of Health	**Clinical guidelines** for healthcare providers on how to manage HIV patients in healthcare settings including protocols of HIV testing and treatment and interactions of routine drugs with HIV medication.
**PEPFAR Country Operational Plan (COP)**	Country division of PEPFAR	**Annual report** at the country level detailing strategic and financial plans to manage HIV/AIDS in the coming fiscal year.

We excluded documents that focused exclusively on other communicable diseases apart from HIV (e.g., tuberculosis, sexually transmitted diseases), however, if a country had a common policy document for these conditions and HIV, they were included. Additionally, for countries that did not have a national-level PEPFAR COP, we included Region Operational Plans (e.g., for Nepal, we included PEPFAR’s Asia Regional Operational Plan).

### Document collection and search criteria

We collected publicly available documents from the official websites of the WHO, U.S. State Department, and national Ministries of Health. For documents not available at these websites, we conducted targeted internet-based searches in English using a public search engine (search string: “[country name]” + “HIV” + “policy” + “[document type]”: PDF). We also contacted USAID country directors via email to request documents we were unable to source via online searches. All included documents were saved in PDF format and archived in a public repository [27].

### Identification of NCDs and NCD risk factors

We searched the included documents for mentions of screening and/or treatment of one or more of four NCDs within an HIV care setting (diabetes, hypertension, mental health conditions including depression, and cervical cancer) and/or two NCD risk factors (alcohol use and tobacco use). These conditions were selected because they represent the leading causes of NCD-related morbidity and mortality in LMICs [1].

### Identifying integration strategies

We used the WHO’s Building Blocks framework to identify and classify health system-strengthening strategies related to screening and/or treatment of the selected NCDs within HIV care settings (S1 Table) [15]. The framework specifies six building blocks–health service delivery, health workforce, health information systems, access to essential medicines (including vaccines and technologies), health systems financing, and leadership and governance–that enhance system capacity to deliver high quality care. Several studies have illustrated the value of the building blocks in supporting improvements in health system performance [17,28,29].

### Coding instrument

Our coding instrument was developed using Qualtrics, a web-based survey development software. Similar instruments have been used in prior HIV policy reviews, such as the UNAIDS Checklist and Reference List for Developing and Reviewing a National Strategic Plan for HIV, which systematically assesses national HIV guidance [25].

We extracted two major categories of data: (1) screening and treatment of selected NCDs and/or NCD risk factors and (2) health-system strengthening strategies (organized under the WHO building blocks) when mentioned in relation to an NCD and/or NCD risk factor screening and/or treatment. All variables used binary response options (“yes” or “no”) to record the presence of relevant data under each category. Each category included open-ended text fields to capture potentially relevant data that did not fit predefined codes.

Using the instrument, we first collected metadata on document type (country or global), country name, publisher, year and any mentions of selected NCDs in the document (yes/no). If no NCDs or NCD risk factors were mentioned, the survey terminated automatically and did not allow further data collection on that document. If one or more NCD was identified, coders proceeded to extract information on NCD screening and/or treatment content, followed by NCD-related health system-strengthening strategies.

For screening and treatment items, we developed subcodes for each NCD and NCD risk factor with illustrative examples to guide the coders. For example, screening subcodes included “Screening for cervical cancer (e.g., VIA, HPV DNA, pap smear)” and treatment subcodes included “Treatment for tobacco use (e.g., brief counseling, group counseling, referral to Quitline, NRT)” with yes/no response options. The category of mental health conditions included references to depression, anxiety, suicidal ideation, mood disorders, mental confusion, and psychological distress, which captured all mentions of mental health-related symptoms across the documents.

For items related to health system-strengthening strategies, we used the WHO’s six building blocks as primary codes and developed subcodes informed by the WHO’s definitions of each building block as well as expert input from the study team [15]. These subcodes captured specific strategies defined by the WHO to strengthen health systems. When mentioned in the documents in the context of NCD screening and/or treatment, we coded “yes” to indicate the presence of that strategy. For example, under the service delivery code, subcodes included strategies such as “improve coordination of NCD services with other parts of the healthcare system”, “ensure availability of NCD screening equipment”, and “launch or support community outreach for NCD screening” [15].

### Data extraction and analysis

To ensure coding consistency, data extraction was completed in two phases. First, four authors pilot-tested the coding instrument on 18% of included documents (n = 12) and resolved discrepancies through discussion. Findings from this phase informed revisions to the coding instrument. Authors then worked in rotating pairs to double code the remaining documents. Discrepancies were resolved through discussion and unresolved items were adjudicated through group consensus with a senior author (DS). Inter-rater reliability was calculated prior to consensus using percent agreement. Percent agreement was calculated on 44 documents (65% of included documents).

The included documents are policy plans and guidelines with limited narrative detail on implementation context. Accordingly, we used a quantitative content analysis approach [30]. This method involves grouping extracted text into predefined categories and calculating the frequency and distribution of each category across the dataset. As specified above, we organized our data into two analytic categories: screening and treatment of selected NCDs and NCD risk factors, and health system-strengthening strategies based on the WHO building blocks. We conducted a summative analysis by counting the number of “yes” responses for each category across all documents using the final consensus-coded dataset. All analyses were conducted in Microsoft Excel.

Free-text responses that did not fit predefined categories were grouped under “other screening method,” “other treatment method,” or “other strategy.” Free-text responses accounted for less than 10% of all coded data. Each response was reviewed during consensus meetings, and when appropriate, recategorized under existing codes to preserve analytical coherence. Less than 2% of free-text responses remained in the “other” categories in the final dataset.

## Results

We analyzed a total of 68 documents. This included 66 documents from 22 countries (40% of 54 PEPFAR-supported LMICs) and the 2021 WHO Consolidated Guidelines on HIV Prevention, Testing, Treatment, Service Delivery, and Monitoring and the 2023 PEPFAR Country and Regional Operational Plan Guidance and Technical Considerations. Most countries were located in Africa (n = 15, 68.2%), followed by East Asia (n = 4, 18.2%), South Asia (n = 2, 9.1%) and Latin America (n = 1, 4.5%) (S2 Table). Inter-rater reliability across coder pairs was high, with a mean percent agreement of 95.9% (range: 92.0–97.6%).

Ten documents from eight countries (15.1%) did not mention NCDs in general or by the specific disease condition. Laos was the only country whose National Technical Guidelines lacked any mention of NCDs; four countries’ National Strategic Plans (Liberia, Laos, Nepal, Botswana) and five countries’ PEPFAR Operating Plans (Cambodia, Liberia, Sierra Leone, Ghana, Myanmar) also did not mention NCDs.

### Screening and treatment of NCDs in HIV policy

The WHO Guidelines and 56 of the 66 country documents mentioned screening and treatment for all six selected NCDs and NCD risk factors in the context of HIV care. In contrast, the PEPFAR Plan discussed screening and treatment for hypertension, and screening for cervical cancer and mental illness among PWH. Table 2 summarizes the prevalence of screening and treatment mentions by disease condition in the 66 country-level documents. Cervical cancer was the most frequently mentioned NCD under screening (72.7%). Mental health conditions had the most frequent number of mentions under treatment (68.2%). Tobacco use received the least number of mentions for both screening (15.2%) and treatment (24.2%).

**Table 2:** Percent of country documents that mentioned NCD screening and treatment by NCD and NCD risk factor (n = 66).

Disease condition	Screening N (%)	Treatment N (%)
Cervical Cancer	48 (72.7)	40 (60.6)
Mental health conditions	34 (51.5)	45 (68.2)
Hypertension	29 (43.9)	31 (47.0)
Diabetes	27 (40.9)	28 (42.4)
Alcohol Use	19 (28.8)	21 (31.8)
Tobacco Use	10 (15.2)	16 (24.2)

### Health system-strengthening strategies relevant to selected NCDs

In the PEPFAR and WHO documents, the most frequently cited strategies were in the categories of service delivery (e.g., establishing clear guidelines for NCD screening and treatment within HIV care) and leadership and governance (e.g., adopting policy and regulations that jointly address the NCD-HIV burden). Table 3 presents the frequencies of health system-strengthening strategies in the 66 country documents, organized by the WHO building blocks. Strategies related to ***service delivery*** were cited most frequently. The most common service delivery strategy was coordinating NCD services with other parts of the healthcare system (74.2%). Over a third of documents (34.8%) described guidelines and protocols for NCD screening and treatment in HIV care settings and 24.2% mentioned establishing referral systems between HIV and NCD services. Strategies related to ***health workforce*** were found in approximately one-third of documents. The most frequently mentioned strategy was training HIV healthcare staff in NCD management (33.3%). Other notable health workforce-related strategies included developing system-level support tools for healthcare staff (21.2%), creating opportunities for staff mentorship and knowledge sharing (18.1%) and offering financial incentives to HIV staff for performing additional NCD-related tasks (18.1%). ***Leadership and governance*** strategies were also common, particularly the adoption of global guidance related to NCDs and HIV (28.8%) and leadership-driven collaborations to promote integration (19.7%).

**Table 3:** Distribution of health system-strengthening strategies across country documents organized by the WHO Building Blocks (n = 66).

Health system-strengthening strategies organized by the WHO Building Blocks	Country Documents (n = 66), n (%)
**Health Service Delivery**	
Coordination of NCD services with other parts of the healthcare system	49 (74.2%)
Establish guidelines and protocols for NCD screening and treatment	23 (34.8%)
Launch or support community outreach to screen HIV patients for NCDs	16 (24.2%)
Ensure availability of screening equipment for NCDs	11 (16.7%)
Ensure availability of treatment equipment for NCDs	6 (9.1%)
Conduct research to inform integration of NCDs	5 (7.6%)
Other strategies (e.g., collaboration with religious leaders)	11 (16.7%)
**Health Workforce**	
Availability of training in NCD management for healthcare staff	22 (33.3%)
System-level support for healthcare staff (e.g., toolkits)	14 (21.2%)
Established mentorship and knowledge sharing for staff related to NCD management	12 (18.1%)
Financial incentives offered for staff to offer NCD care	12 (18.1%)
Adequate number of staff specialized in combined HIV-NCD care	5 (7.6%)
Staff prioritizes treating NCDs	4 (6.1%)
Other strategies (e.g., increase clinical supervision)	9 (13.6%)
**Health Information Systems**	
Upgraded HIV health records with NCD information	7 (10.6%)
Deploy technologies for managing NCD patients in HIV care (e.g., text messaging)	7 (10.6%)
Introduce regular performance feedback mechanisms	3 (4.5%)
Increase investment in health information systems	0
Training for HIV staff on using integrated record systems	0
Other strategies (e.g., strengthen NCD surveillance in national monitoring systems, introduce telemedicine services)	4 (6.1%)
**Access to Essential Medicines**	
Ensure availability of HPV vaccines for cervical cancer prevention in HIV care	14 (21.2%)
Provide clinical guidelines to address drug interactions between HIV and NCD medications	13 (19.7%)
Establish or strengthen supply chains for NCD medication and supplies	3 (4.5%)
Other strategies (e.g., case-by-case costing for NCD drugs, include NCD drugs on essential HIV drug list)	5 (7.6%)
**Health Systems Financing**	
Increase allocation of funds for NCD-related management in HIV programs	8 (12.1%)
Reduce out-of-pocket costs for patients	6 (6.1%)
Develop collaborations focused on integrating NCD in HIV care (e.g., public-private partnerships, engage donors)	2 (3.0%)
Develop a financing system for HIV and NCD integration	1 (1.5%)
Other strategies (e.g., introducing program-specific budget controls to prioritize NCD services)	6 (6.1%)
**Leadership and Governance**	
Adopt global policies and regulations for HIV-NCD integration	19 (28.8%)
Strengthen multi-sectoral collaboration for HIV-NCD integration	13 (19.7%)
Political priority towards HIV-NCD integration	10 (15.2%)
Other strategies (e.g., design strategic plans for HIV-NCD integration)	3 (4.5%)

Strategies under the ***access to essential medicines*** domain were less frequently mentioned. These included making HPV vaccines for cervical cancer prevention available in HIV clinics (21.2%) and providing clinical guidelines to manage NCD-HIV drug interactions (19.7%). Strategies related to ***health information systems*** and ***health systems financing*** were rarely mentioned. Information systems strategies included upgrading HIV health records to include NCD information (10.6%) and using technologies such as text messaging to remind patients about NCD screening (10.6%). The most frequently mentioned financing strategy involved increasing funds for NCD management within HIV programs (12.2%).

During coding, we identified infrequent mentions of challenges related to delivering NCD services within HIV care. The WHO Guidelines mentioned challenges that could be mapped to the building blocks of service delivery (e.g., difficulty generating research evidence for care integration), health workforce (e.g., increased workload for HIV providers) and health system financing (e.g., lack of funds from HIV donors for NCD management). Among country documents, the most frequently mentioned challenge was the lack of healthcare staff to provide joint HIV-NCD care (10.6%; health workforce building block). This was followed by limited coordination of NCD services with other parts of the healthcare system (6.1%) and lack of availability of NCD treatment equipment (6.1%), both mapped to the service delivery building block (S3 Table). The PEPFAR Plan did not include any mentions of challenges to implementing NCD prevention and treatment services.

## Discussion

Over 80% of the documents reviewed mentioned NCD screening and/or treatment in the context of HIV care. However, the emphasis placed on screening and treatment of specific NCDs varied, with cervical cancer and depression/psychological distress receiving the most number of mentions. Further, we found that health system-strengthening strategies that were mentioned in relation to NCD screening and/or treatment within HIV care most frequently focused on improving service delivery and developing the health workforce, while health system financing and governance-related strategies were less frequently reported.

Global guidance documents from the WHO and PEPFAR frame integration of NCD services within HIV care as a strategic priority to improve the long-term delivery of care for PWH [5,11]. This policy focus is reflected in our analysis. We found that the WHO HIV Prevention Guidelines referenced all six NCDs and NCD risk factors and included a range of health system-strengthening strategies to increase the provision of NCD services in HIV care settings. In contrast, the PEPFAR Plan prioritized screening and treatment of mental illness, cervical cancer and hypertension, which is aligned with PEPFAR’s funding priorities and 2025 Vision [11,31,32].

An emphasis on cervical cancer and mental health conditions was reflected across 56 documents from PEPFAR-supported countries. This is likely due to sustained global investments and multilateral initiatives to scale evidence-based cervical cancer interventions. Examples include large public-private partnerships, such as GoFurther, and the adoption of WHO’s 2020 Global Strategy to eliminate cervical cancer by 194 countries [31,33]. In March 2024, stakeholders at the world’s first Global Cervical Cancer Elimination Forum committed USD 600 million in new funding to expand HPV vaccination coverage [34]. These funding commitments and policy emphasis are consistent with the substantially higher burden of cervical cancer among women living with HIV, who are six times more likely to develop cervical cancer compared with women without HIV, and the classification of invasive cervical cancer as an AIDS-defining illness [35,36]. This may explain why LMICs in our analysis highly prioritized diagnosing and treating cervical cancer within HIV systems.

Similarly, the frequent mentions of screening and treatment for mental health conditions reflect priorities set by global agencies. Increasingly, the broad push to recognize mental health as an integral part of universal health coverage has extended to global HIV policy, with global organizations recognizing the need for integrating mental health screening and psychosocial support into HIV interventions [12,37]. In 2022, WHO and UNAIDS published joint guidance that highlights task sharing and community-based screening as approaches to expand mental health services for PWH [38]. Together, these developments suggest that mental health integration into HIV services is well-supported at the global policy level, which may explain its consistent inclusion by LMICs in their national policies.

In contrast, behavioral risk factors of alcohol and tobacco use were the least prominent across included documents. This omission is striking considering long‑standing global commitments to address these risk factors. Over 183 countries covering 90% of the world’s population have ratified the WHO Framework Convention on Tobacco Control and adopted a range of tobacco control policies, including services to support tobacco cessation [39,40]. A parallel global commitment exists for alcohol: the WHO’s action plan for alcohol use over 2022–2030 builds on its 2010 Strategy to Reduce the Harmful Use of Alcohol, which was endorsed by 193 countries and provides detailed guidance to curb alcohol‑related harms [41].

Theory-guided research is needed to explore the questions that these documents raise. For example, Atun et al.’s framework suggests that integrating health interventions in different settings is likely to succeed when global priorities, funding streams, institutional capacity, and intervention design are aligned [42]. Cervical cancer and mental health both benefit from this alignment; cervical cancer has dedicated global initiatives and funding streams for PWH, while mental health-HIV integration is supported by published global implementation guidance. Understanding the factors that drive these decisions and the effectiveness of strategies that advance NCD-HIV integration is critical to achieving global priorities to improve health outcomes for PWH.

Our analysis also identified a range of health-system strengthening strategies that were being employed in LMICs to increase NCD screening and treatment within HIV care. The service delivery-based strategy of coordinating NCD services with other parts of the healthcare system appeared in 75% of documents, followed by a third of documents reporting training HIV staff in NCD management. We were not able to identify models for service integration that these documents reference. However, a recent scoping review of studies evaluating models to integrate HIV/TB care with NCD prevention and treatment in LMICs found that referral-based and co-location-based integration models were the most common, with fewer studies evaluating fully integrated models where HIV and NCD systems and workflows were shared [43]. The review also found that provider training was the most commonly used strategy to implement integrated interventions [43]. Such approaches may be more feasible in LMIC contexts because they require smaller investments and minimal changes to existing HIV infrastructure [7,16]. At the same time, this means that country efforts to integrate care remain concentrated on service delivery and workforce strategies, whereas sustained integration requires system-level reforms that are tailored to country context and can create changes in governance and financing mechanisms [5,16].

Only 10% of documents mentioned health system-strengthening strategies related to health information systems, in spite of WHO’s emphasis on robust monitoring and reporting systems to support NCD-HIV integration [5]. However, this may be due to structural and financial constraints within LMIC health systems, where investments in digital infrastructure compete with immediate service delivery needs and where HIV electronic medical record systems are standalone and lack interoperability with national health information systems [16,17,19,21].

In this analysis, as in prior studies, the WHO Building Blocks proved to be a useful analytic framework for evaluating the extent to which national policy documents mention NCD management within HIV care systems [17,28,29]. Countries are already using this and other frameworks, such as the Framework for Integrated People Centered Care, to guide systems improvements [5]. Efforts to accelerate HIV-NCD integration may be supported by expanding the application of these frameworks in these settings and specifying these strategies within national strategic plans. Consistent application of standard frameworks would also facilitate monitoring and evaluation and cross‑country comparisons to generate examples of effective approaches for achieving integrated HIV‑NCD care.

Historically, PEPFAR and other global HIV initiatives have supported the development of HIV programs that expanded access to antiretroviral therapy and strengthened HIV health infrastructure [4,11,13]. However, these systems were organized primarily to support HIV care and were not designed to deliver comprehensive primary care or manage multiple chronic conditions. Recent changes in global HIV funding streams may affect HIV care delivery systems in the future. Regardless of these changes, our findings provide a baseline description of the system-level strategies that LMICs are using to support integrate NCD-HIV integration, which offer useful insights for future efforts to integrate HIV, NCD, and primary care services.

This analysis had several limitations. First, we analyzed documents that were publicly available at the time of data collection (2023–2024), and some countries may have since released updated policies or operational plans. Second, although our analytic focus on PEPFAR-supported countries enabled cross-country and global comparison, this focus may limit the generalizability of findings to non-PEPFAR settings. In addition, we were unable to obtain complete sets of English-language documents for all PEPFAR-supported countries and therefore excluded six countries from the analysis (Barbados, Vietnam, Trinidad and Tobago, Philippines, Thailand, and Togo), which may further limit representativeness across certain regions and policy contexts. Third, our descriptive analysis focused on the presence or absence of policy mentions for selected NCDs and risk factors, without evaluating factors that influence policy adoption. It is possible that interventions are underway in countries but are not reflected in national policy documents. Finally, as mentioned above, we acknowledge that reductions in PEPFAR and UNAIDS funding announced in 2025 may affect future efforts to integrate NCD services within HIV care systems, including efforts to transition from vertically organized HIV programs toward more integrated chronic care systems. These developments occurred after the data collection period and caution should be exercised when interpreting the current relevance of policy documents included in this analysis.

## Conclusion

Consistent with global WHO and PEPFAR guidance, HIV policy documents indicate a commitment to integrating NCD services in the context of HIV care. However, there is variation in terms of which NCDs are prioritized, which ultimately effects resource allocation. Strategies for integrating NCD prevention and treatment are currently concentrated in service delivery and health workforce development with less emphasis on financing, governance, and health information systems. This again, may reflect decisions that align with country resources to expand access to NCD-related care to meet population health priorities. Strengthening NCD-HIV integration will require improved information systems to monitor progress and drive improvements, enhanced supply chains to increase access to essential medications and sustained investment in implementing WHO guidelines for screening and treatment of NCD risk factors. To advance stated goals for HIV-NCD integration, future research is needed to identify factors that either drive or constrain investments in integrating NCD services within HIV care settings. This need is especially urgent given recent shifts in U.S. foreign assistance priorities since 2025, which may reshape national HIV policies and guidelines and influence how countries approach the integration of HIV and NCD services.

## Supporting information

S1 Table Definitions of World Health Organization Building Blocks. (DOCX)

S2 Table List of countries included in the analysis by World Bank-designated world regions. (DOCX)

S3 Table Prevalence of barriers to NCD-HIV integration in country documents organized by the WHO Building Blocks (n = 66). (DOCX)
