# Supplementary material for: A content analysis of global and national policies, plans, and guidelines to integrate NCDs with HIV care in LMICs
Source: PLOS Glob Public Health. 2026 Aug 7;6(8):e0007072. doi: 10.1371/journal.pgph.0007072 (PMC13450756; doi:10.1371/journal.pgph.0007072)
Supplement: Supplementary file 1 [file pgph.0007072.s001.docx]

**S1 Table. Definitions of World Health Organization Building Blocks [1].**

| **WHO Health System Building Block** | **Definition** |
| --- | --- |
| Health service delivery | The timely, efficient, and patient-centered provision of health services that achieve desired health outcomes with minimal wastage of resources. |
| Health workforce | A workforce that is adequate and fairly distributed across a population, and that is competent, responsive, and productive to achieve the best possible health outcomes. |
| Health information systems | The responsible generation, collection, and analysis of data on health status, determinants, and health-system performance, and timely dissemination of data to facilitate health system planning and decision making. |
| Access to essential medicines | Assuring the quality, safety, and efficacy of essential medical products, vaccines, and technologies that are cost-effective and equitably accessible. |
| Health systems financing | The availability of adequate funding and provision of stewardship of resources to ensure people in need can use health services and remain protected from financial catastrophe associated with healthcare payments. |
| Leadership and governance | A government that ensures the design and application of strategic policy frameworks that adhere to system design principles and account for effective oversight, regulation, accountability, and coalition building. |

**Reference:**

1. World Health Organization. Monitoring the Building Blocks of Health Systems: A Handbook of Indicators and Their Measurement Strategies: World Health Organization; 2010 [Available from: https://iris.who.int/bitstream/handle/10665/258734/9789241564052-eng.pdf].
