# Supplementary material for: A content analysis of global and national policies, plans, and guidelines to integrate NCDs with HIV care in LMICs
Source: PLOS Glob Public Health. 2026 Aug 7;6(8):e0007072. doi: 10.1371/journal.pgph.0007072 (PMC13450756; doi:10.1371/journal.pgph.0007072)
Supplement: Supplementary file 2 [file pgph.0007072.s002.docx]

**S2 Table. List of countries included in the analysis by World Bank-designated world regions.**

| **Region** | **Country** |
| --- | --- |
| **Asia and Pacific** | Burma  Cambodia |
|  | Lao PDR |
|  | Papua New Guinea |
| **Eastern and Southern Africa** | Botswana |
|  | Eswatini |
|  | Ethiopia |
|  | Kenya |
|  | Lesotho |
|  | Malawi |
|  | Namibia |
|  | Rwanda |
|  | South Africa |
|  | Uganda |
|  | Zambia |
| **Western and Central Africa** | Ghana |
|  | Liberia |
|  | Nigeria |
|  | Sierra Leone |
| **Latin America and the Caribbean** | Jamaica |
| **South Asia** | India |
|  | Nepal |
