# Supplementary material for: A content analysis of global and national policies, plans, and guidelines to integrate NCDs with HIV care in LMICs
Source: PLOS Glob Public Health. 2026 Aug 7;6(8):e0007072. doi: 10.1371/journal.pgph.0007072 (PMC13450756; doi:10.1371/journal.pgph.0007072)
Supplement: Supplementary file 3 [file pgph.0007072.s003.docx]

**S3 Table. Distribution of challenges related to delivering NCD services within HIV care in country documents, organized by the WHO Building Blocks (n=66).**

| **Challenges in delivering NCD care in HIV settings organized by the WHO Building Blocks** | **Country Documents**  **n=66, n (%)** |
| --- | --- |
| **Health Service Delivery** |  |
| Lack of coordination of NCD services with other parts of the healthcare system | 4 (6.1) |
| Lack of guidelines and protocols for NCD screening and treatment | 2 (3.0) |
| Lack of community outreach to screen HIV patients for NCDs | 1 (1.5) |
| Lack of screening equipment for NCDs | 2 (3.0) |
| Lack of treatment equipment for NCDs | 4 (6.1) |
| Lack of research to inform integration of NCDs | 2 (3.0) |
| Other barriers (e.g., travel restrictions on clinic visits) | 2 (3.0) |
| **Health Workforce** |  |
| Lack of training in NCD management for healthcare staff | 0 |
| Lack of staff expertise or skill in NCD management | 2 (3.0) |
| Lack of mentorship programs for staff related to NCD management | 1 (1.5) |
| Lack of remuneration for staff to offer NCD care | 0 |
| Lack of healthcare staff specialized in combined HIV-NCD care | 7 (10.6) |
| Lack of staff motivation to treat NCDs | 2 (3.0) |
| Other barriers (e.g., high staff turnover) | 5 (7.6) |
| **Health Information Systems** |  |
| Lack of health records that document NCD information | 0 |
| Lack of technologies for managing NCD patients in HIV care (e.g., SMS text messaging) | 0 |
| Lack of performance feedback mechanisms | 0 |
| Lack of investment in health information systems | 0 |
| Lack of training for HIV staff on using integrated record systems | 0 |
| Other barriers (e.g., incomplete data reporting from health centers) | 1 (1.5) |
| **Access to Essential Medicines** |  |
| Lack of HPV vaccines for cervical cancer prevention in HIV care | 2 (3.0) |
| Lack of clinical guidelines to address drug interactions between HIV and NCD medications | 3 (4.5) |
| Absence of or poor supply chain for NCD medication and supplies | 2 (3.0) |
| Other barriers (e.g., high cost of NCD medications) | 1 (1.5) |
| **Health Systems Financing** |  |
| Lack of funding for NCD-related management in HIV programs | 3 (4.5) |
| High out-of-pocket costs for patients | 1 (1.5) |
| Lack of collaboration with donors | 0 |
| Lack of a financing system for HIV and NCD integration | 1 (1.5) |
| Other barriers | 0 |
| **Leadership and Governance** |  |
| Lack of global policies and regulations for HIV-NCD integration | 0 |
| Lack of multi-sectoral collaboration for HIV-NCD integration | 3 (4.5) |
| Lack of political priority towards HIV-NCD integration | 0 |
| Other barriers (e.g., lack of leadership accountability in promoting NCD-HIV integration) | 1 (1.5) |
